# Mineral Waters in Malarial Diseases

**Published:** 1887-01

**Authors:** 


### MINERAL WATERS IN MALARIAL DISEASES.

The value of mineral waters in disease is no longer questioned ; it is universally recognized. Hippocıates was said to have been a firm believer in its efficacy. In America, in early times, it is known that the Indians made yearly pilgrimages to certain "healing springs," and to this day the reputation of certain of them is still maintained. There is, in particular, one near Ocean Springs, Mississippi. of whose healing powers marvelous tales are told. That certain waters will cure and thoroughly eradicate malaria from the system there is abundant testimony; and the enterprize of the genus "Yankee" has demonstrated that in order to get the beneficial effect of medicinal water, it is not necessary to go to the Springs, but the water may be, and daily is, transported in bottles and barrels to great distances, without losing its curative property.

The Massanetta Springs, situated in the beautiful Shenandoah Valley of Virginia, near Harrisonburg, at the foot of the Massanutten mountain, has, for more than fifty years, borne a reputation as an anti-malarial water ; and from an analysis of its water, lately made by Professor Mallett, of the University of Virginia, it is to be readily seen that the reputation is well founded. Not only for malaria is it useful, but for the thousand and one sequellæ that follow the invasion of this element into the blood. According to this analysis, the water contains, besides 86.67 per cent. (to the gallon) of nitrogen gas, and 8.44 of oxygen, (escaping in bubbles) the following solid, constituents: calcium carbonate 14.778, magnesium 6.949, *iron* (ferrous) .375, sodium 1.228, manganese, lithium, ammonium-chloride, potassium cloride, potassium sulphate, *arsenious*

*oxide* (in salt), *phosphoric acid,* silica, alumina, etc., in decided quantities.

Dr. Billings having pointed out the extensive prevalence(?) of malaria in the South, it behoves us to call attention to a remedy; and we suggest to our physicians, especially those in the bottoms, to give the subject of mineral waters investigation, and to try the virtues of the Massanetta, so universally extolled. Mr. Shafer authorizes us to say that he will deliver a barrel of this water, "free on board," for $5.00, to any physician in Texas who feels disposed to give it a trial. We all know that there occur cases of chronic malarial toxæmia that are the despair of the physician, and on which quinine seems to have no effect. Here is an opportunity that should not be lost. It is said that a short time after the close of the war a petition, signed by six of our Major Generals and fourteen Brigadier-Generals, was presented to Congress, asking the purchase of the Massanetta Springs, with a view of establishing a Military and Marine Hospital, being described as "decidedly restorative in that class of chronic diseases which entail so much persistent ill-health and discomfort upon the officers and men of the armies and navies." Unfortunately for the sailor and soldier, but little attention was paid to the request, and the Massanetta Springs are still private property, and are now beyond price.

